# Case Report: Neonatal-onset parkinsonian-pyramidal syndrome caused by novel compound heterozygous *FBXO7* variants

**DOI:** 10.3389/fped.2026.1934625

**Published:** 2026-09-17

**Authors:** Weicong Pu, Zelei Liu, Yanyan Cao, Jiancheng Jiao, Min Sun, Li Ma

**Affiliations:** 1 Department of Neonatology, Children’s Hospital of Hebei Province, Shijiazhuang, China; 2 Pediatric Clinical Research Centre of Hebei Province, Children's Hospital of Hebei Province, Shijiazhuang, China; 3 Genomic Medicine Research Laboratory, Children’s Hospital of Hebei Province, Shijiazhuang, China

**Keywords:** *FBXO7*, hypertonia, neonate, parkinsonian-pyramidal syndrome, whole-exome sequencing

## Abstract

**Background:**

Early-onset parkinsonism is often associated with genetic factors. Pathogenic variants in the *FBXO7* gene are a rare cause of early-onset parkinsonism, typically presenting as parkinsonian-pyramidal syndrome (PPS), also known as Parkinson's disease-15 (PARK15). To date, only three cases of infantile-onset PARK15 have been reported worldwide. Herein, we describe a Chinese patient with neonatal-onset PARK15, who carried novel compound heterozygous *FBXO7* variants.

**Case presentation:**

A 37-hour-old male infant with generalized limb rigidity was referred for evaluation of neonatal-onset hypertonia. Physical examination revealed abnormal vocalization, poor sucking and swallowing (requiring nasogastric tube feeding), and abdominal muscle rigidity. Pyramidal involvement was evidenced by positive Babinski sign and its equivalents, together with hyperreflexia, while extrapyramidal findings included lead-pipe rigidity of all four limbs with markedly increased muscle tone. Whole-exome sequencing identified two compound heterozygous nonsense variants in *FBXO7*, c.376G > T (p.Gly126*) and c.1033C > T (p.Arg345*), inherited from the father and mother, respectively. Both variants were classified as pathogenic. Following diagnosis, the parents elected to discontinue active in-hospital treatment, and the infant was discharged home. Nevertheless, a trial of oral levodopa was initiated at home. Despite levodopa treatment, the infant showed no clinical improvement and died at 89 days of age.

**Conclusions:**

To our knowledge, this is the first reported case of neonatal-onset PARK15 caused by *FBXO7* variants. This case expands the mutational and clinical spectrum of PARK15 in the neonatal period.

## 1 Introduction

Parkinson's disease (PD), the second most common neurodegenerative disorder after Alzheimer's disease, typically manifests at a median age at onset of 60 years. Although most cases are idiopathic, approximately 10% have been linked to pathogenic variants in more than 20 genes involved in the pathogenesis of PD and related conditions (1, 2). Among these, pathogenic variants in *FBXO7* (3) cause a rare autosomal recessive form of neurodegeneration known as Parkinson's disease-15 (PARK15) or parkinsonian-pyramidal syndrome (PPS).

In 2008, Shojaee et al. first identified variants in *FBXO7* in a consanguineous Iranian family (4). Subsequent studies have identified multiple pathogenic homozygous and compound heterozygous *FBXO7* variants (5–7). These variants, clustered within regions encoding the N- and C-terminal domains of the *FBXO7* protein, are associated with a broad spectrum of clinical manifestations. The age of onset varies considerably, ranging from infancy to adulthood. To date, 21 families with PARK15 comprising 39 patients have been reported worldwide. However, only three infantile-onset cases have been documented, with the youngest onset at 5 months of age (8, 9).

To the best of our knowledge, no neonatal cases of PARK15 associated with *FBXO7* variants have been reported previously. Herein, we describe a Chinese infant with neonatal-onset PARK15 who carried compound heterozygous variants in the *FBXO7* gene.

## 2 Case presentation

In December 2024, a 37-hour-old male infant with limb hypertonia was transferred to our department from a local hospital. He was born at 40 weeks and 2 days of gestation via elective cesarean section to a G2P1 mother. His birth weight was 3,930 g, his Apgar score was 9, and there was no evidence of perinatal asphyxia. Immediately after birth, he exhibited severe limb rigidity and a weak cry. The patient was admitted to the local neonatal unit and treated with penicillin and intravenous immunoglobulin for thrombocytopenia. However, his hypertonia did not improve, and he was transferred to our center for further management. The parents were healthy and nonconsanguineous, and there was no significant family history of inherited disorders. The mother's first pregnancy resulted in a missed abortion at 8 weeks of gestation, and fetal chromosomal analysis revealed trisomy 22.

On admission, physical examination revealed decreased responsiveness and no crying in response to stimulation. Numerous petechiae were observed on the chest and abdomen. The anterior fontanelle was flat; however, mild neck stiffness was noted. The infant responded to supraorbital pressure and had an intact pupillary light reflex. Ocular examination showed no nystagmus, no limitation of extraocular movements, and no ptosis. Facial examination revealed no facial droop, symmetric nasolabial folds, and complete eyelid closure. The patient's vital signs remained stable. Abdominal examination revealed abdominal rigidity with no hepatosplenomegaly and normal bowel sounds. Primitive reflexes were absent. Pyramidal involvement was evidenced by positive Babinski sign and its equivalents, together with hyperreflexia, while extrapyramidal findings included lead-pipe rigidity of all four limbs with markedly increased muscle tone.

Blood gas analysis showed normal results (pH: 7.419, PaCO_2_: 38.2 mmHg, PaO_2_: 78.6 mmHg, HCO_3_^−^: 24.7 mmol/L, base excess: +0.4 mmol/L). Serum electrolytes, lactate, and blood glucose were also within normal limits. A complete blood count revealed a white blood cell count of 15,100/mm^3^ and a platelet count of 39,000/mm^3^, whereas the C-reactive protein level was 1.85 mg/L. Serum magnesium, ammonia, and homocysteine, as well as liver, renal, and thyroid function, were within normal limits. Immunological evaluation was positive for the anti-Ro52kD antibody and antinuclear antibodies (ANA), with a titer of 1:100 and a nuclear speckled pattern, whereas anti-platelet antibodies were absent. Routine and biochemical analyses of the cerebrospinal fluid were within normal limits. Cranial magnetic resonance imaging revealed symmetrical T1 hyperintensities in the bilateral globi pallidi and thalami (Figure 1A) along with heterogeneous signals in the transverse, sigmoid, and superior sagittal sinuses (Figures 1B,C). Video-electroencephalography (vEEG) revealed multifocal discharges of spikes, sharp waves, spike-and-wave complexes, and sharp-and-slow-wave complexes (Figure 2). No electrographic seizures or clinical epileptic events were recorded.

**Figure 1:**
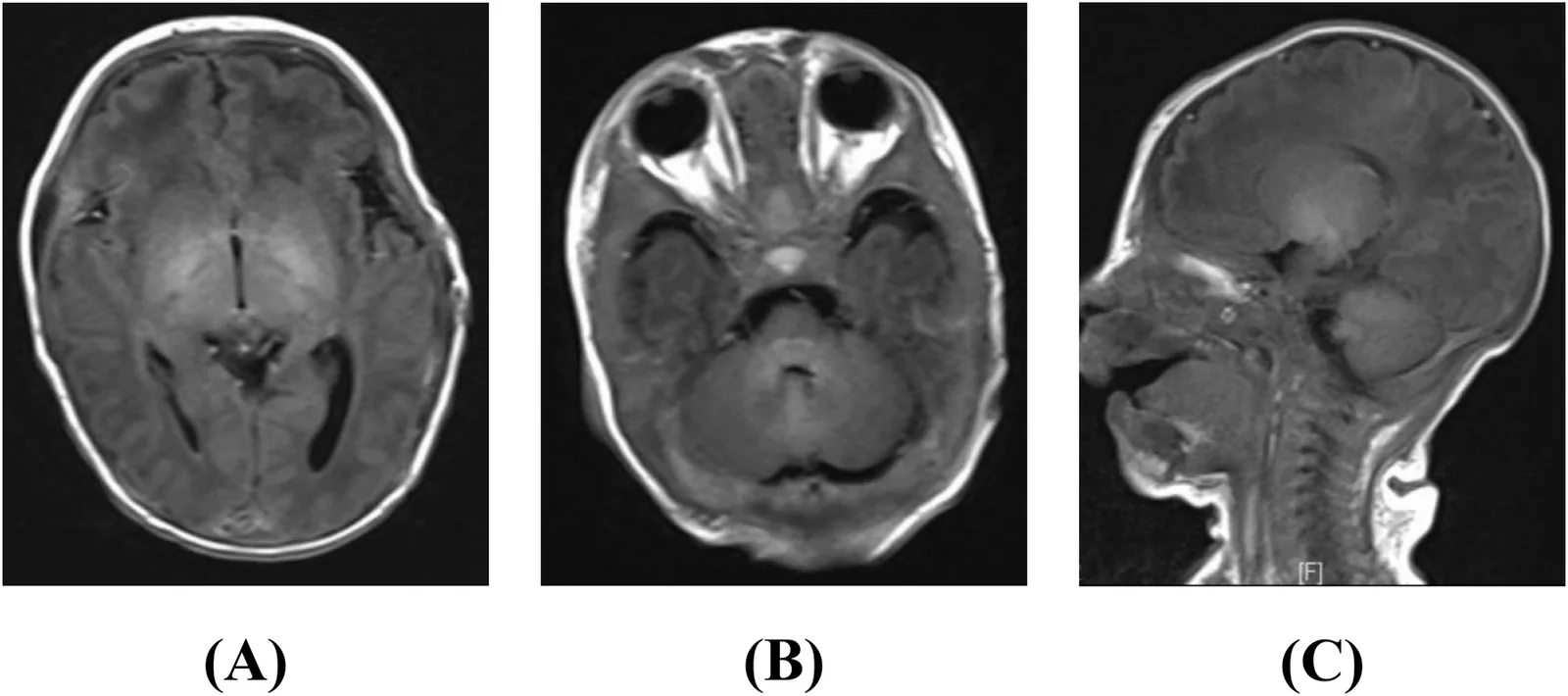
Cranial magnetic resonance imaging of the infant. **(A)** There were symmetrical T1 hyperintensities in the bilateral globi pallidi and thalami. **(B)** and **(C)** There were heterogeneous signals in the transverse, sigmoid, and superior sagittal sinuses.

**Figure 2:**
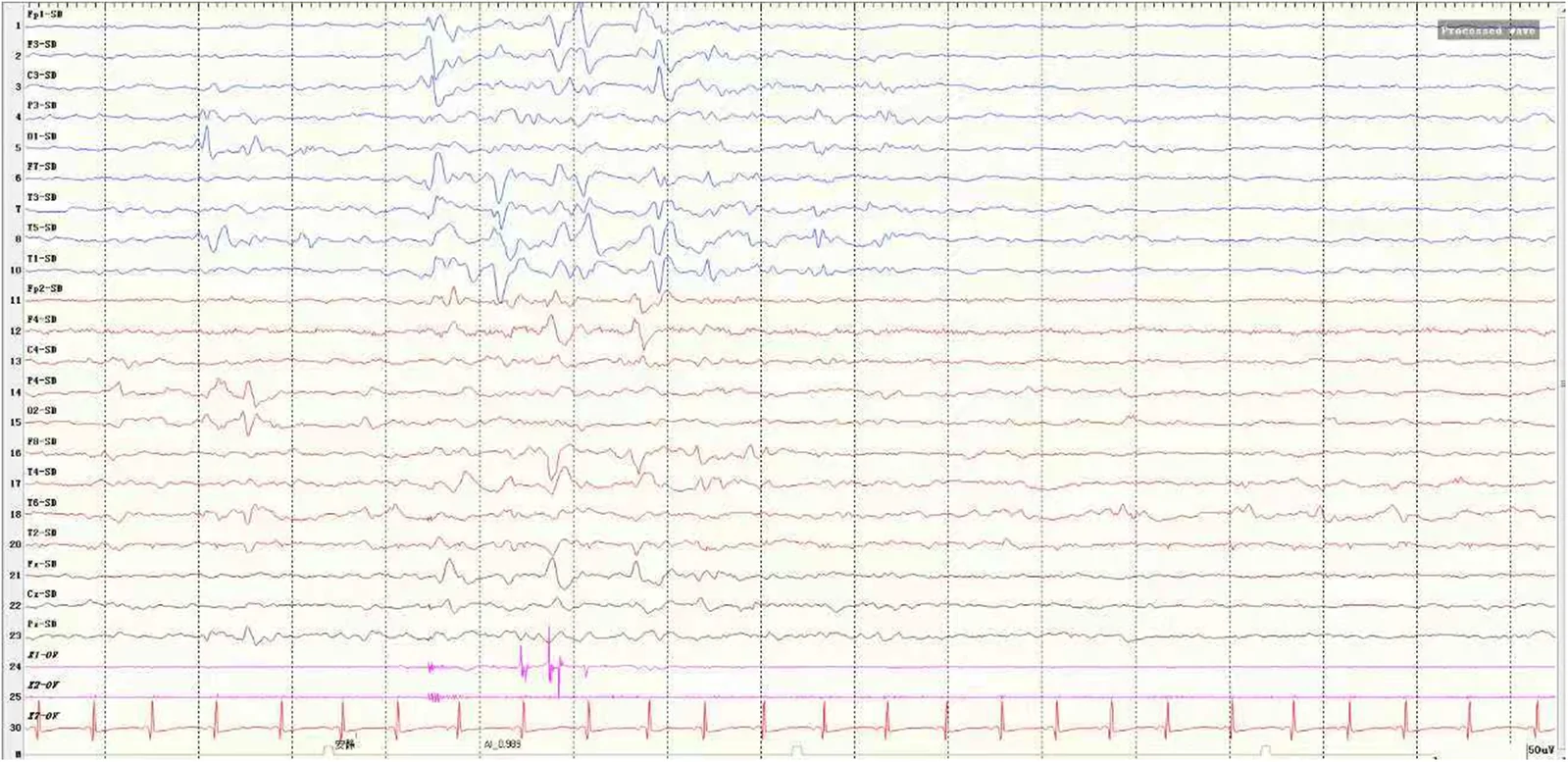
Video-electroencephalography (vEEG) tracing of the infant. vEEG revealed multifocal discharges of spikes, sharp waves, spike-and-wave complexes, and sharp-and-slow-wave complexes. No electrographic seizures or clinical events were captured.

After admission, the infant received oxygen via nasal cannula and piperacillin-tazobactam. Because neonatal seizures could not be excluded, phenobarbital, midazolam, and vitamin B6 were administered. Immune thrombocytopenia was suspected, prompting administration of intravenous immunoglobulin, after which the platelet count normalized on follow-up. Despite standard care, the infant exhibited persistent axial and limb rigidity, severe hypertonia, and absent rooting and sucking reflexes, necessitating nasogastric tube feeding. Neurological examination revealed no crying in response to stimuli and abnormal vocalization. Given this clinical phenotype and the maternal history of an adverse pregnancy outcome, whole-exome sequencing was performed after obtaining parental consent.

Whole-exome sequencing (WES) performed by Beijing Fulgent Technologies Inc. (Beijing, China) identified two novel compound heterozygous variants in *FBXO7* (NM_012179.4): c.376G > T (p.Gly126*) in exon 2 and c.1033C > T (p.Arg345*) in exon 7. Sanger sequencing confirmed paternal and maternal inheritance of these variants (Figure 3). According to the American College of Medical Genetics and Genomics guidelines, both nonsense variants were classified as pathogenic [PVS1 + PM2 + PM3 (Supporting)]. A diagnosis of PARK15 was ultimately established based on the clinical presentation, including hypertonia, limb rigidity, dysphagia, and abnormal vocalization, together with the genetic test results.

**Figure 3:**
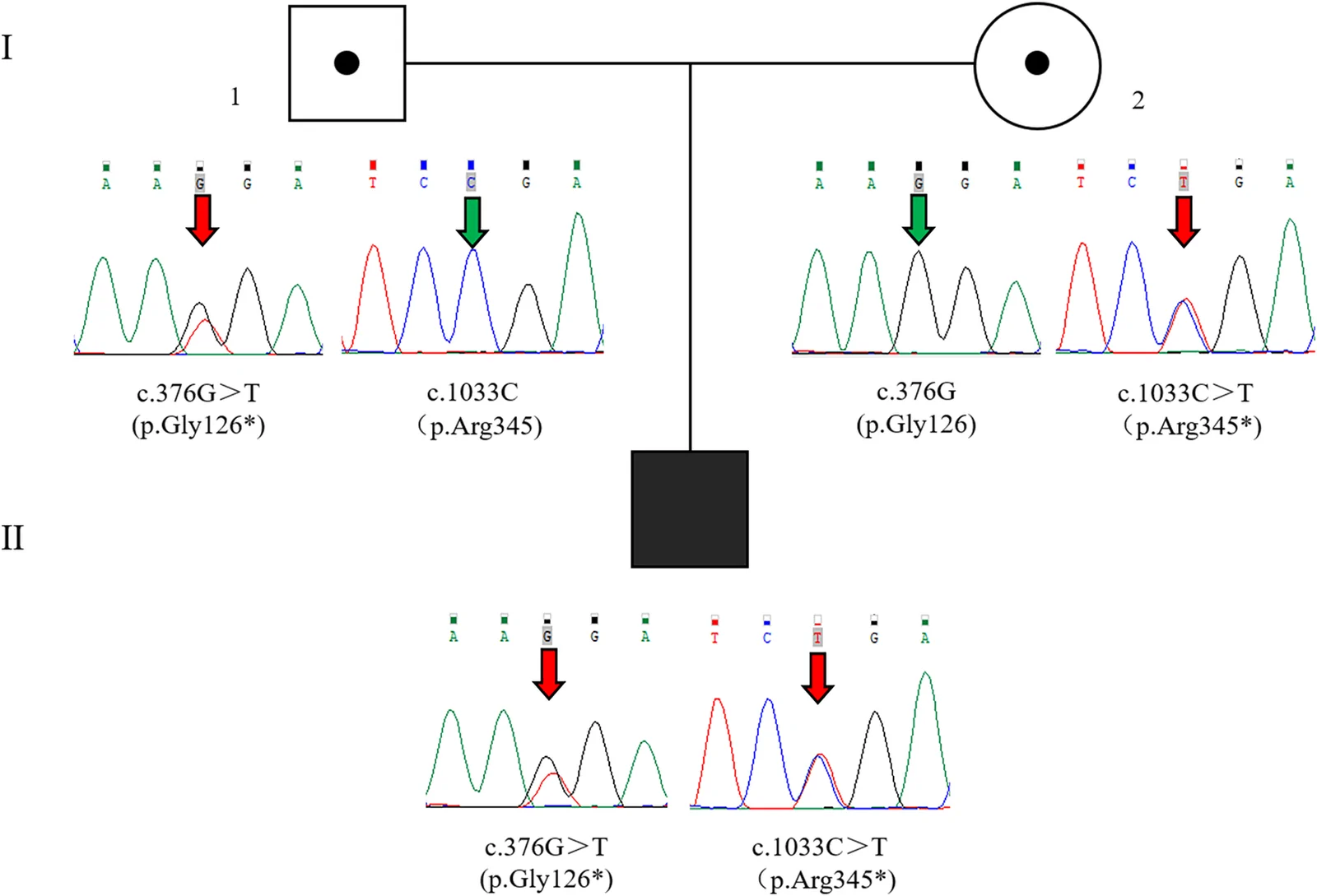
Family pedigree and *FBXO7* variants confirmed using Sanger sequencing. Patient (II) had compound heterozygous variants c.376G > T (p.Gly126*) and c.1033C > T (p.Arg345*), which were inherited from his father (I-1) and mother (I-2), respectively.

After 28 days of inpatient therapy without clinical improvement, the parents elected to discontinue active in-hospital treatment (including nasogastric tube feeding, routine nursing care and continuous monitoring) because of the presumed poor prognosis, and the infant was discharged home. Nevertheless, given the family's wish to attempt oral pharmacological therapy, oral levodopa was initiated at home on day 30 of life at 3 mg/kg/day, administered in three divided doses. The dose was increased by 3 mg/kg/day every 5 days, reaching the target maintenance dose of 15 mg/kg/day in three divided doses on day 50. Treatment was continued for approximately 2 months, until the patient's death on day 89. Throughout the treatment period, no clinical neurological improvement was observed: the patient remained unable to suck or swallow (requiring ongoing nasogastric feeding), and limb rigidity and hypertonia persisted unchanged. Outpatient assessments on days 43 and 71 confirmed the absence of clinical response. Follow-up laboratory findings were unremarkable. The infant died on day 89 from feeding-related asphyxia.

## 3 Discussion and conclusions

We report a case of neonatal-onset PARK15 associated with novel compound heterozygous nonsense variants in *FBXO7* (c.376G > T, p.Gly126* and c.1033C > T, p.Arg345*). The patient presented with refractory limb rigidity that did not respond to levodopa therapy, and both *FBXO7* variants were nonsense. To our knowledge, this is the first reported case of neonatal-onset *FBXO7*-related PARK15.

The *FBXO7* gene is located on chromosome 22q12–q13, spans approximately 24.15 kb, and contains nine exons. It encodes the *FBXO7* protein, which comprises 522 amino acids and contains multiple functional domains (10). Pathogenic variants in *FBXO7* disrupt key cellular processes and the maintenance of normal dopaminergic neuronal function, thereby contributing to PARK15. *FBXO7* is broadly expressed in human tissues and exhibits prominent expression in brain motor regions, including the extrapyramidal and pyramidal systems, which is consistent with the clinical manifestations of PARK15 caused by loss-of-function *FBXO7* variants. In zebrafish, *FBXO7* loss leads to dopaminergic dysfunction and motor impairment (11). *FBXO7* knockout mice show reduced proteasomal activity, early motor deficits, and premature death (12). *FBXO7* mutations contribute to PARK15 progression by inducing mitochondrial dysfunction, impaired mitophagy, and altered proteasomal activity (9, 12).

To date, 21 families with PARK15 (including the present family) have been reported worldwide, comprising 39 patients (23 males and 16 females) (4–9, 13–22). PARK15 has a broad age at onset, ranging from the neonatal period to 52 years, with a peak between 10 and 30 years, three presented with clinical symptoms after 40 years of age (14, 21), and four presented with clinical signs within the first year of life (8, 9). Most families had a history of consanguinity. The core clinical phenotype of PARK15 combines parkinsonian features with pyramidal signs, although pyramidal involvement is absent in some families (7, 17, 22). Additionally, a variety of nonclassical symptoms have been reported, including dysphagia, dysarthria, oculomotor abnormalities, choreiform movements, REM sleep behavior disorder, intellectual developmental delay, marked preference for sweet foods, and seizures (7, 13, 14, 16, 19, 21, 22), highlighting the phenotypic diversity of the disorder. Most patients showed improvement in motor symptoms with levodopa therapy, although adverse effects, including gastrointestinal reactions, aggressive behavior, impulse control disorders, and neuropsychiatric symptoms, were frequently reported (16–18, 20). In the present case, the infant's clinical presentation was consistent with PPS. Nevertheless, we acknowledge that classical parkinsonian features, such as resting tremor, bradykinesia, and postural instability, cannot be reliably evaluated or unequivocally demonstrated in a neonate. The diagnosis of PARK15 in this patient therefore rests primarily on the identification of biallelic pathogenic *FBXO7* nonsense variants, together with a nonspecific neonatal phenotype. No improvement was observed following levodopa administration, possibly reflecting the early-onset and severe phenotype. Wang et al. (20) reported that, compared with missense *FBXO7* variants, nonsense, frameshift, and splice-site variants are associated with an earlier age at onset. Our patient presented in the neonatal period, and both mutations in *FBXO7* were nonsense.

Only three cases of infantile-onset PARK15 have been reported worldwide, with the youngest being diagnosed at 5 months of age. Jin et al. (8) described two Chinese siblings carrying compound heterozygous *FBXO7* variants. The older sibling, a full-term infant with an unremarkable perinatal history, developed hypertonia and was unable to sit independently by 6 months of age. Initially misdiagnosed with cerebral palsy, he responded poorly to rehabilitation and could not walk at 6 years of age, exhibiting persistent hypertonia, limb rigidity, speech impairment, and intellectual disability. Similarly, his younger brother showed hypertonia and cognitive delay from 6 months of age and died at 2 years owing to severe pneumonia. Al Rawi et al. (9) reported a 5-month-old female infant with reduced limb movements and truncal hypotonia, which later progressed to limb rigidity and fluctuating dystonia. Optic atrophy was noted at 2.5 years, and global developmental delay was evident at 5 years. A homozygous *FBXO7* variant was identified in that study. The patient initially responded to levodopa with improved mobility; however, the benefits diminished over time. In our case, the neonate exhibited limb rigidity and hypertonia from birth. Initially, neonatal seizures were suspected and symptomatic treatment, including anticonvulsants and vitamin B6, was initiated, but no improvement was observed. Common causes, such as hypoxic-ischemic encephalopathy, intracranial hemorrhage, infection, electrolyte disturbances, hypoglycemia, and bilirubin encephalopathy were subsequently excluded through comprehensive investigation. A 4-hour continuous vEEG recording demonstrated multifocal discharges of spikes, sharp waves, spike-and-wave complexes, and sharp-and-slow-wave complexes. No ictal electrographic changes or clinical epileptic events were captured throughout monitoring. The persistent muscular tension showed no temporal correlation with discharges, which did not support an epileptic origin. In addition, the following genetic and metabolic disorders were systematically considered and excluded: maple syrup urine disease and organic acidemias (absence of maple syrup odor, normal blood gas, ammonia, and lactate, and unremarkable blood and urine metabolic screening); urea cycle disorders (normal ammonia, unremarkable metabolic screening); mitochondrial disorders including Leigh syndrome (normal lactate, absence of characteristic lesions on MRI); and tyrosinemia type I (no elevation of succinylacetone on urine organic acid analysis). Other genetic etiologies, including early-onset parkinsonism-dystonia (ATP13A2, PLA2G6, PRKN, PINK1, and ATP1A3), pyridoxine-dependent epilepsy (ALDH7A1), and early infantile epileptic encephalopathy (KCNQ2, SCN2A, KCNT1, PACS1), were excluded through comprehensive analysis of WES data. The diagnosis of PARK15 was established based on the identification of biallelic *FBXO7* pathogenic variants and familial segregation analysis. All four patients with infantile-onset PARK15, including the present patient, had poor outcomes (Table 1).

**Table 1:** FBXO7 variations in patients with infantile-onset PARK15.

Infant number (Ref.)	Case 1 (8)	Case 2 (8)	Case 3 (9)	Case 4 (This study)
Reported year	2020	2020	2024	2025
Sex	M	M	F	M
Age at onset	6 months	6 months	5 months	After birth
Age at last assessment	6 years	2 years	5 years	89 days
Perinatal history	Unremarkable	Unremarkable	Unremarkable	Unremarkable
Parental consanguinity	NA	NA	Yes	No
Country of origin	China	China	UK	China
Clinical presentation	Increased muscle tension and could not sit independently	Increased muscle tension and poor cognitive development	Reduced limb movements, truncal hypotonia, and pyramidal syndrome progressed to limb rigidity and fluctuating dystonia.	Persistent rigidity, hypertonia, abnormal vocalization, and dysphagia
Cranial MRI	Unremarkable	Unremarkable	Thinning of corpus callosum	Symmetrical T1 hyperintensity in the bilateral globus pallidus and thalamus, heterogeneous mixed signals in the transverse, sigmoid, and superior sagittal sinuses
Levodopa response	NA	NA	Yes	No
Outcome	Could not walk independently, had increased muscle tone and limb stiffness, could not speak, and had intellectual developmental delays at 6 years	Died from severe lung infection	Motor impairment, moderate global developmental delay, developmental quotient is 40 at 5 years	Died from feeding-related asphyxia at 89 days
Mutation type	Compound heterozygous	Compound heterozygous	Homozygous	Compound heterozygous
Mutation at the nucleotide level	c.402G > A and c.872-1G > A	c.402G > A and c.872-1G > A	c.749T > C	c.376G > T and c.1033C > T
Mutation at the protein level	p.Trp134* and IVS5-1G > A	p.Trp134* and IVS5-1G > A	p.Leu250Pro	p.Gly126* and p.Arg345*

F, female; M, male; MRI, magnetic resonance imaging; NA, data not available.

Approximately 80% of the nearly 7,000 known rare diseases are monogenic disorders (23), many of which are life-threatening or disabling. The majority currently lack effective treatments, imposing severe burdens on affected families. In the present case, PARK15 was inherited in an autosomal recessive pattern, with each parent carrying a heterozygous pathogenic FBXO7 variant, conferring a 25% risk of recurrence in each pregnancy. For future pregnancies, prenatal diagnosis or preimplantation genetic testing could be considered to reduce the risk of recurrence.

This report describes a rare case of neonatal-onset PARK15 manifesting initially as limb rigidity and hypertonia. This presentation highlights the need to consider PARK15 in neonates with persistent rigidity, hypertonia, abnormal vocalization, and dysphagia, in whom early genetic testing may facilitate a definitive diagnosis. This case broadens the understanding of the mutational and phenotypic spectrum of PARK15 caused by *FBXO7* pathogenic variants.

## Data Availability

The datasets presented in this article are not readily available because the parents provided written informed consent only for publication of the anonymised clinical and molecular findings, not for public release of the underlying sequencing reads. Requests to access the datasets should be directed to the corresponding author.
